# New directions in cellular therapy of cancer: a summary of the summit on cellular therapy for cancer

**DOI:** 10.1186/1479-5876-10-48

**Published:** 2012-03-15

**Authors:** David F Stroncek, Carolina Berger, Martin A Cheever, Richard W Childs, Mark E Dudley, Peter Flynn, Luca Gattinoni, James R Heath, Michael Kalos, Francesco M Marincola, Jeffrey S Miller, Gustavo Mostoslavsky, Daniel J Powell, Mahendra Rao, Nicholas P Restifo, Steven A Rosenberg, John O'Shea, Cornelis JM Melief

**Affiliations:** 1 Department of Transfusion Medicine, Clinical Center, NIH, Bethesda, USA; 2 Fred Hutchinson Cancer Research Center, Seattle, USA; 3 Fred Hutchinson Cancer Research Center and Cancer Immunotherapy Trials Network (CITN), Seattle, USA; 4 National Heart, Lung, Blood Institute, NIH, Bethesda, USA; 5 Surgery Branch, National Cancer Institute, NIH, Bethesda, USA; 6 Fate Therapeutics, Inc,, San Diego, USA; 7 California Institute of Technology, Pasadena, USA; 8 University of Pennsylvania, Philadelphia, USA; 9 Center for Human Immunology, NIH, Bethesda, USA; 10 University of Minnesota, Minneapolis, USA; 11 Boston University School of Medicine, Boston, USA; 12 Center for Regenerative Medicine, NIH, Bethesda, USA; 13 National Institute of Arthritis and Musculoskeletal and Skin Diseases, Bethesda, USA; 14 Leiden University Medical Center and Immune System Activation, Leiden, The Netherlands

## Abstract

A summit on cellular therapy for cancer discussed and presented advances related to the use of adoptive cellular therapy for melanoma and other cancers. The summit revealed that this field is advancing rapidly. Conventional cellular therapies, such as tumor infiltrating lymphocytes (TIL), are becoming more effective and more available. Gene therapy is becoming an important tool in adoptive cell therapy. Lymphocytes are being engineered to express high affinity T cell receptors (TCRs), chimeric antibody-T cell receptors (CARs) and cytokines. T cell subsets with more naïve and stem cell-like characteristics have been shown in pre-clinical models to be more effective than unselected populations and it is now possible to reprogram T cells and to produce T cells with stem cell characteristics. In the future, combinations of adoptive transfer of T cells and specific vaccination against the cognate antigen can be envisaged to further enhance the effectiveness of these therapies.

## Background

A summit on cellular therapy for cancer was held on November 1 and 2, 2011 at the NIH in Bethesda, Maryland. Advances related to the use of adoptive cellular therapy for melanoma and other cancers were presented and discussed and are summarized in this review.

## Tumor infiltrating lymphocytes (TIL)

Treatment of melanoma with tumor infiltrating lymphocyte (TIL) cells of high functional avidity has been improved by treating patients with lymphocyte reducing chemotherapy prior to the administration of the TIL cells. Preconditioning the TIL recipient with non-myeloablative chemotherapy has resulted in a objective clinical response rate of 49% with 13% complete responses and preconditioning with non-myeloablative chemotherapy plus 12 Gy total body irradiation (TBI) further improved objective clinical response rate to 72% with 40% complete responses [1].

Several advancements associated with manufacture of TIL have been made and as a result TIL production has become more practical. Traditionally, initial TIL cultures were screened for reactivity to tumor antigens and only the reactive cultures were selectively expanded. Many cell processing laboratories are no longer screening initial TIL colonies for tumor-reactive cells, rather, they are expanding all isolated TIL cells based on the initial finding that nearly 80% of TILs possess autologous tumor reactivity [2] This has reduced the duration of time that TIL spend in culture and as a result these cells are known as "young" TIL. Clinical studies are evaluating whether that young TIL are as effective as TIL produced using traditional methods [3].

TIL have been traditionally manufactured using tissue culture plates for initial culture and flasks and bags for rapid expansion protocols (REP). This process, however, results in large final culture volumes: 30 to 60 liters. These large volumes are associated with the use large quantities of media, cytokines, and additives. In addition, harvesting these large volumes is time consuming. In order to simplify and reduce the quantity of reagents and labor associated with TIL production, two new methods are being tested for TIL REP; bioreactors and gas permeable flasks. The WAVE bioreactor can be used for TIL REP [4]. The volume of the final TIL product is reduced by the WAVE bioreactor, but the WAVE requires the investment of capital and specialized staff training. The volume of media required for TIL REP can also be reduced by using gas permeable flasks [5]. The flasks are simple to use and do not require capital investment. In addition, gas permeable flasks can also be used for initial TIL culture.

The increased clinical effectiveness and improved production methods are leading to the more widespread use of TIL to treat patients with melanoma.

## Engineered T cells

While TIL Therapy is effective, melanoma samples cannot be obtained for TIL production from all patients and, in some cases, TIL cannot be isolated from the resected tumor. Engineered T cells are being used increasingly for patients from whom TIL are not available. Two general approaches involving engineered T cells are being used clinically. Both involve the use of autologous peripheral blood T cells; one involves gene transfer of high affinity T cell receptors (TCR) and the other gene transfer of chimeric antibody-T cell receptors (CAR) [6].

Patients with melanoma have been treated with T cells engineered using recombinant retroviral vectors to express HLA-2 restricted high affinity T cell receptors (TCRs) specific for melanoma antigens MART-1 and gp100 [7,8]. While patients treated with these engineered autologous cells have had objective clinical responses, some patients have experienced autoimmune responses due to the destruction of normal melanocytes in the skin, eyes and ears [8]. Another adoptive cellular therapy approach utilizing engineered T cells involves the use of TCRs specific for cancer testis antigens that are expressed by fetal tissue and cancer, but not by adult cells, such as NY-ESO-1. NY-ESO-1 is expressed by 10 to 50% of metastatic melanomas, 80% of synovial cell sarcomas and breast, prostate, thyroid and ovarian cancers [6]. TCRs specific for NY-ESO-1 have been used to treat patients with melanoma and sarcoma and have resulted in objective clinical responses in 5 of 11 melanoma patients and 4 of 6 synovial sarcoma patients [9]. Protocols are also being developed that involve gene transfer of vectors encoding IL-12 and MAGE-A3 specific TCRs.

Another approach involves the transduction of autologous T cells to express CARs made up of the variable region a tumor specific antibody fused to an intracellular signaling domain capable of activating T cells. Typically, a CAR is comprised of an extracellular scFv portion of a monoclonal antibody and an intracellular CD3 zeta chain in tandem with a co-stimulatory signaling domain, such as CD28. In addition, some CARs include other stimulatory factors such as 4-1BB or OX-40, alone or in combination with CD28 [6]. Since CARs have the specificity of a monoclonal antibody, they are not HLA restricted and they can be used to treat any patient whose tumor expresses the antigen to which the monoclonal antibody is directed. Autologous T cells engineered to express anti-CD19 CAR have been effective in treating patients with lymphoma and chronic lymphocytic leukemia (CLL) [10-13]. CD19 CAR T cell adoptive therapy has resulted in dramatic clinical responses which have been associated with in vivo expansion and long term persistence of the engineered T cells. Some patients have experienced tumor lysis syndrome and prolonged depletion of B cells is common.

A clinical protocol that uses the autologous T cells expressing CAR specific for the folate receptor -alpha (FRα)[14] is being developed by University of Pennsylvania investigators in cooperation with the National Cancer Institute (NCI) Cancer Immunotherapy Trial Network (CITN). FRα is over expressed on the surface of epithelial malignancies including ovarian, breast, renal, colorectal, lung, and other solid cancers, but its expression is limited on normal tissue. The protocol involves adoptive cell therapy with genetically engineered autologous T cells given to patients with ovarian cancer following lymphodepletion alone or followed by the administration of recombinant IL-7 and was rationalized by the established role for IL-7 in maintaining T cell memory and homeostasis, as well as initial observations by Powell et al. that transferred tumor antigen-specific T cells dramatically up-regulate the IL-7 receptor immediately after infusion [15].

## Reprogramming cells

The reprogramming of adult cells in order to produce more primitive cells or stem cells is becoming an important part of cellular therapy of cancer. Adult cells can be reprogrammed to produce induced pluripotent stem cells (IPSC) which have properties similar to embryonic stem cells. Investigators are now working to reprogram T cells to produce "stem-like" T cells that are more effective in adoptive cell therapy.

### Induced pluripotent stem cells

Methods to reprogram stem cells have improved greatly since Yamanaka first demonstrated that the transfer of 4 transcription factors, Oct4, Klf4, Sox2 and cMyc, into fibroblasts can produce IPSCs [16]. IPSCs differ in some respects from embryonic stem cells (ESCs) but these differences can be reduced by removing the transcription factor used for reprogramming. One method involves reprogramming using a single excisable lentivral vector containing all 4 transcription factors which allows for highly efficient reprogramming and IPSCs free of exogenous transgenes using from fresh and store blood samples [17-19]. Traditional culture of IPSCs involves the growth of cells on feeder cell layers or extracellular matrix derived from animals and the use of media supplemented with animal serum. Methods are being developed to produce and culture IPSC using xenogenic free materials and reagents which will improve the safety of these products. Companies are developing platforms for high throughput IPSC generation. These platforms also allow for cell maintenance and characterization.

### Reprogramming T cells

Several studies have found that T cell phenotype affects their effectiveness for adoptive cell therapy. Comparison of TIL cells from patients responding to therapy and those that did not has found that clinical responses were associated with TIL that expressed co-stimulatory molecules CD27 and CD28, have longer telomeres and persist longer in vivo. Several investigators have been exploring methods to produce cytotoxic T cells which persist longer and are more effectively clinically [1]. Using CMV reactive T cells and a macaque model, Carolina Berger and Stan Riddell found that CMV-specific effector CD8^+ ^T cell (T_E_) populations derived from central memory T cells (T_CM_) rather than effecter memory T cells (T_EM_) retained the ability to survive long-term in the circulation, bone marrow, and lymph nodes [20]. Of note, the T_CM_-derived T_E _cells differentiated to both T_CM _and T_EM _phenotypes *in vivo *and responded efficiently to antigen challenge [20]. This work has recently been extended to human virus-specific T cells [21].

A major new area reviewed at the meeting was the idea that mature, post-thymic lymphocytes have stem cell-like qualities. Restifo et al. have recently found that Th17-polarized CD4^+ ^T cells have stem cell-like qualities. Th17 have superior anti-tumour activities than their Th1 counterparts, are resistant to apoptosis and persist long-term after adoptive cell transfer. Most importantly, they have the stem cell-like properties of self-renewal and multipotency [22].

In addition, Gattinoni et al. have identified a subpopulation of circulating T cells with both naïve and memory T cell properties with a CD45RO-, CCR7+, CD45RA+, CD62L+, CD27+, CD28+ and IL-7Rα+ phenotype which they have called stem central memory T (Tscm) cells [23]. These T scm cells have greater proliferative potential, longer in vivo survival and are more potent for adoptive cell transfer than naïve, central memory, effector memory or effector T cells [23,24].

While Tscm cells are potentially very effective in adoptive cellular therapy, very few Tscm cells are present in the circulation. Several laboratories have been investigating methods to reprogram T cells in order to produce the large quantities of Tscm cells that would be needed for adoptive cell therapy. Wnt signaling/β-catenin and mTor signaling pathways have been found to be important in T cell maturation [25,26]. The Wnt/β-catenin pathway is activated in naïve T cell, but becomes progressively less active as T cells mature. Because the Wnt/β-catenin pathway is important in cancer, a number of drugs are being developed that interact with this pathway. Gattinoni et al. have found that Tscm cells can be efficiently generated *in vitro *when naïve T cells are stimulated in the presence of a Wnt pathway activator, TWS119 [23,24]. In the future, it may be possible to use similar methods to generate large quantities of Tscm cells *ex vivo *for use in adoptive cell therapy coupling TCR or CAR engineering with pharmacological modulation of T cell differentiation.

## Vaccine therapy using long peptides

An alternative to adoptive transfer of T cells is vaccination with DCs loaded with short tumor peptides that bind exactly to specific HLA epitopes, however the effectiveness of these therapies have been limited by insufficiently consistent and robust effector T cell responses. Vaccination with longer tumor peptides (28-35 amino acids) results in more efficient peptide processing and presentation than short peptides. The treatment of 20 women with high grade vulvar intraepithelial neoplasia with 3 subcutaneous Human Papilloma Virus 16 (HPV 16) E6 and E7 synthetic peptide vaccines resulted in clinical responses in 15 of 19 patients at 12 months of follow-up [27]. HPV 16-specific-T cell responses were significantly greater in the group of patients with complete regression of their lesions compared to the non-responders.

## NK cells

Autologous NK cells are being expanded *ex vivo *by 100-1000 fold and used to treat patients with CLL, colon cancer and renal cell carcinoma (RCC). Patients are first treated with the proteasome inhibitor bortezomib to increase tumor sensitivity to NK cell cytotoxicity mediated by TNF-related apoptosis-inducing ligand (TRAIL) prior to infusion of expanded autologous NK cells with low dose subcutaneous IL-2 administered twice daily for 1 week following infusion [28]. Phase I dose escalation of increasing numbers of adoptively transferred autologous NK cells continues, with 2 infusions of up to a dose of 1 × 10^8 ^NK cells/kg having already been established to be safe, with preliminary evidence for anti-tumor effects being observed against tumors such as RCC and CLL.

Allogeneic NK cells are being used to treat hematological malignancies. These allogeneic NK cells protocols make use of *in vivo *expansion by using pretreatment lymphoreduction therapy and post-infusion IL-2 therapy [29]. As an alternative to IL-2, the CITN recently developed a clinical trial to test the safety and efficacy of outpatient IL-15 therapy in order to stimulate NK and CD8+ T cells. IL-15, compared to IL-2, may enhance cell-based immunotherapy as it is hypothesized to have less of an effect on suppressive regulatory T-cells that down-regulate NK cell and T cell function. This may lead to better clinical efficacy and has broad implications for the field of immunotherapy.

## Evaluation of biomarkers for adoptive cellular therapies

A critical part of the treatment of cancer with adoptive cellular therapies is the monitoring of recipients following treatment. Clinical trials of cellular therapies for cancer should include biomarker studies in an integrated, quality, supported, and meta-analyzeable manner [30]. For T cell therapy clinical trials, the biomarker classes assessed should evaluate T cell presence, biologically relevant phenotypes and functions of the T cells, T cell bioactivity, as well as recipient immune responses to the infused T cells. Numerous approaches can be used to evaluate each of these classes of biomarkers [31]. These principals were recently applied in a clinical trial which treated CLLwith anti-CD19 T cells and this approach provided a remarkable breadth and depth of information concerning T cell persistence, phenotype, and function [12,13].

## Regulatory

Most clinical studies involving adoptive cellular therapies are conducted under an Investigational New Drug (IND) application. FDA encourages early preparation for critical points in the IND process, such as moving into initial clinical studies and transitioning to pivotal clinical trials. Proper preparation allows for an easier transition, a better designed study, and a higher likelihood of success. FDA staff encourages sponsors and investigators to take advantage of formal meetings, such as pre-IND meetings, and is often willing to speak with sponsors and investigators through direct informal interactions. In addition, numerous FDA websites contain information useful for investigators (http://www.fda.gov/biologicsbloodvaccines/newsevents/ucm232821.htm and http://www.fda.gov/BiologicsBloodVaccines/GuidanceComplianceRegulatoryInformation/OtherRecommendationsforManufacturers/ucm094338.htm).

## Conclusions

The field of adoptive cell therapy is advancing rapidly. Conventional cellular therapies, such as TIL, are becoming more effective and more available. Gene therapy is becoming an important tool in adoptive cell therapy. Autologous lymphocytes are being engineered to express TCRs, CARs and cytokines. T cell subsets with more naïve and stem cell-like characteristics have been shown in pre clinical models to be more effective than unselected populations. In the future combination of adoptive transfer of T cells and specific vaccination against the cognate antigen can be envisaged to further enhance the effectiveness of treatment.
